# Errata

**Published:** 1903-05

**Authors:** 


Errata.—We regret very much the occurrence of several errors in the splendid paper by Dr. H. K. Leake in our April number. It is almost unpardonable in me that even his name was misprinted. But everybody knows Dr. Henry K. Leake, of Dallas. Dr. J. C. Anderson's name was also given as "J. C. Andrews," and, in my March number, the name of Dr. J. W. Kenney appeared as "Kennedy." Oh! my, I'm awful sorry. I'll have to kill that proof reader at the office, and then go out and kick myself.

